# Disparities in the level of COVID-19 health literacy and the associated factors among employees in long-term care facilities in Taiwan

**DOI:** 10.1186/s12913-023-09721-z

**Published:** 2023-06-28

**Authors:** Lan-Ping Lin, Jia-Rong Yu, Jin-Ding Lin

**Affiliations:** 1 Institute of Long-term Care, MacKay Medical College, New Taipei City, Taiwan; 2 Ren-Ai Senior Citizens’ Home, New Taipei City Government, New Taipei City, Taiwan

**Keywords:** Coronavirus disease 2019 (COVID-19), Health literacy, Caregiving manpower, Elderly welfare agencies, Long-term care

## Abstract

**Background:**

Coronavirus disease 2019 (COVID-19) poses a significant threat to the safety of residents in long-term care facilities, and the staff of long-term care facilities are essential in the care and prevention of major infectious diseases and therefore require good health literacy to ensure the health of residents. The main objective of this study was to examine the health literacy of staff in long-term care facilities and analyze the factors associated with their COVID-19 health literacy in Taiwan to provide a basis for the response mechanism to emerging infectious diseases.

**Methods:**

A cross-sectional survey with a structured questionnaire by a convenience sample method and to assess the COVID-19 health literacy of caregivers working in long-term care facilities in this study. The COVID-19 health literacy scale was a self-administered scale designed to combine the concept of “health literacy” with the 3 levels and 5 stages of preventive medicine. A total of 385 workers from 10 long-term care facilities were surveyed as the study sample, and the validated questionnaires were statistically analyzed using SPSS version 22.0 statistical software. A multivariate logistic regression model was used to establish the associated factors of the COVID-19 health literacy level.

**Results:**

Overall, the mean COVID-19 health literacy score was 88.7 ± 10.4 (range: 58–105). Using a quartile scale, 92 (23.9%) of the study participants had low health literacy (health literacy score < 82), 190 (49.3%) had average health literacy (health literacy score 82–98), and the remaining 103 (26.8%) had good health literacy (health literacy score 99–105). Statistical analysis revealed significant differences (p < 0.05) in the COVID-19 health literacy score by demographic variables (education, job category, number of daily service users, and training related to infectious disease prevention and control) of the study population. The logistic regression analysis of the COVID-19 health literacy level (> 82 vs. ≤82) showed a significant difference in the study sample by gender (male vs. female, OR = 2.46, 95% CI = 1.15–5.26), job category (nurse practitioner vs. caregiver, OR = 7.25, 95% CI = 2.46–21.44), monthly service hours (> 160 h vs. 40–79 h, OR = 0.044, 95% CI = 0.07–0.97), experience caring for confirmed COVID-19 patients (yes vs. no, OR = 0.13, 95% CI = 0.02–0.98), and training related to infectious disease prevention and control (yes vs. no, OR = 2.8, 95% CI = 1.52–5.15).

**Conclusions:**

This study recommends that facilities provide immediate updated COVID-19 information to staff, especially frontline caregivers, and specifically enhance COVID-19 infection control education training for all facility staff to eliminate health literacy disparities.

## Introduction

Elderly people in long-term care facilities (LTCF) constitute a vulnerable population and have high rates of chronic diseases and infectious disease outbreaks, but this has been largely unrecognized in the public health discourse [1]. According to the Taiwan Ministry of Health and Welfare, as of the end of December 2021, there were 1,081 welfare institutions for elderly people [2]; this large number of institutions may increase the risk of infectious epidemics within institutions if not handled carefully. The elderly population has been greatly affected by COVID-19 [3], and those who living in nursing homes are lacking mobility and live in close proximity with unavoidable contact, are especially vulnerable to higher risks of COVID-19 infection [4]. The many clusters of COVID-19 outbreaks that have occurred in and outside of LTCF show that most residents are older, have multiple chronic diseases, experience limitations to physical activity, or have invasive catheter placement; in addition, residents of such facilities are frequently admitted and discharged from medical facilities, which also poses a potential risk of infection. To assist LTCF for elderly individuals in taking appropriate measures, the Taiwan Center for Disease Control has formulated many recommendations and regulations for these settings in response to the COVID-19 epidemic so that they can follow the relevant guidelines to establish correct awareness among the staff and to implement visitor management, health monitoring and notification of abnormalities among staff and service recipients [5].

When elderly residents live in a LTCF where they are at risk of infection, they are more likely to be exposed to COVID-19. General health literacy regarding COVID-19 may be important for attenuating the decline in health-related quality of life by enabling the effective use of health information and adaptive behaviors toward health threats [6]. One study reported that the higher the awareness of infection control is, the better the attitude and behavior of staff in LTCF, and the better they can cooperate with infection control policies [7]. Therefore, it is important for facility staff to follow the relevant guidelines to develop COVID-19 prevention, treatment, and response behaviors, as well as the basic COVID-19 health awareness within LTCF. At this stage, it is important to establish correct knowledge of COVID-19 among the staff of elderly welfare organizations and to ensure the implementation of visitor management and health monitoring of the staff and residents.

Public health entities apply a combination of materials and tools (i.e., webpages, infographics, and videos) when developing health information materials to improve their understandability, actionability, and clarity to improve people’s COVID-19 health literacy [8]. The core of health literacy is health information, and the transmission and communication of health information includes information production, communication processes and the receiving end. In a particular context, the result of health literacy not only is related to the individual’s ability but also depends on the interaction between the individual’s ability and the environment [9]. Some people, including healthcare professionals, often equate health literacy with health education and advocacy in terms of its positioning, but this is not entirely true. Health literacy emphasizes empowerment, i.e., the ability to empower people to become healthier through the enhancement of health literacy. Therefore, all health-related information, whether it concerns food, clothing, housing, transportation, education, or entertainment, falls within the scope of health awareness. The current trend in health literacy is to integrate the medical, public health, and education sectors to achieve a win‒win outcome of two-way health literacy enhancement by using multimedia methods such as cell phones, computers, and the internet to enhance medical professionals’ health literacy and healthy lifestyles [10].

Health literacy is the motivation and ability to access and apply information in complex and situational conditions and to take the right health actions. It is an urgent need for planning a needs-based health literacy programme focusing specifically on COVID-19 literacy [11]. In Australia, McCaffery et al. [12] showed that there are important disparities in COVID-19-related knowledge, attitudes, and behaviors according to people’s health literacy and language. A Japanese study revealed that prevention behaviors were significantly related to health literacy, COVID-19 knowledge, and fear of COVID-19 [13]. Another study in Korea suggested that boosting self-efficacy and the ability to find, evaluate, and apply health information with sufficient evidence from the Internet can improve COVID-19 preventive behaviors [14].

The staff of a geriatric welfare agency should have COVID-19 health literacy not only so that they can understand COVID-19 health information or assist symptomatic residents in seeking medical care but also so that they can locate, understand, and apply relevant knowledge to protect themselves and residents from infection and even develop health behavioral skills to independently perform health behavioral skills. In addition, they should have the motivation and skills to maintain good health behaviors on an ongoing basis. As the COVID-19 epidemic is changing rapidly, the staff of elderly welfare organizations need to be able to keep up with changing COVID-19 information; otherwise, the effectiveness of the organization’s epidemic prevention and management will be affected. However, there are no studies on COVID-19 health literacy and related factors among the staff of elderly welfare organizations in Taiwan. Therefore, this study aims to examine the COVID-19 health literacy of staff in LTCF and analyze its associated factors in Taiwan to provide a basis for the response mechanism of LTCF to emerging infectious diseases.

## Method

This study was reviewed and approved by the Ethical Review Committee of Taipei MacKay Memorial Hospital (No. 21MMHIS379e). According to the Taiwan Ministry of Health and Welfare, as of the end of December 2021, there were 1,078 welfare institutions for elderly individuals, with a caregiver population of 21,944 individuals [15]. The sampling method in this study was based on the online version of Raosoft Inc. software [16]; a sampling error of 5% and 95% confidence interval were set to calculate the number of valid study samples to be 385, which was in line with the originally planned representative sample size. A total of 500 questionnaires were sent out to 6 institutions in the North, 2 in the Central, 1 in the South and 1 in the East, considering geographical differences. Firstly, the researcher verbally explained the purpose, content, target population and relevant procedures of the study to the supervisors of the long-term care institutions and obtained their consent. The researcher visited each LTCF in person, and before conducting the formal questionnaire, the researcher explained the full content of the study to the subjects and obtained their consent to conduct the survey. There were 385 valid questionnaires were collected from 10 LTCF as the study sample, for a valid sample return rate of 77%.

This study employed a structured questionnaire to assess the COVID-19 health literacy of caregivers working in LTCF. The main parts of the questionnaire included the caregiver’s personal and working characteristics and a COVID-19 health literacy scale. The COVID-19 health literacy scale was a self-administered scale designed to combine the concept of “health literacy” with the 3 levels and 5 stages of preventive medicine. Twenty-one questions were included on the scale, and the test subjects scored the questions on a Likert scale (scored from 1 to 5; the higher the score was, the more positive the health awareness of COVID-19). After the initial design of this questionnaire was completed, five experts in the field of public health, academics in the field of long-term care and institutional workers were invited to conduct an expert review in accordance with the purpose of the study, the design and structure of the study and the content of the questionnaire, and the experts were asked to rate the questionnaire according to its suitability. Finally, an expert face validity test was conducted in this study and the results showed that the mean value of the content validity index (CVI) was 0.84, and the questionnaire has good validity. Regarding the reliability of the questionnaire, Cronbach’s alpha coefficient was used to determine the internal consistency of the questionnaire content, with a coefficient of 0.95.

After the questionnaires were completed by the respondents and collected by the researchers, the data were entered into Microsoft Excel software and analyzed by the SPSS 22.0 statistical package for descriptive and inferential statistics. The distribution of demographic variables and COVID-19 health literacy of the staff of the LTCF are presented. For the inferential statistics, independent t-tests, chi-square tests, and one-way ANOVA tests were used to analyze the cross-correlations between the factors and COVID-19 health literacy according to the data attributes. Finally, a logistic regression model was used to establish the associated factors of the COVID-19 health literacy level.

## Results

### Respondents’ demographics and job characteristics

The basic demographic data of the respondents are shown in Tables 1 and 2. The total number of samples in this study was 385; the majority of the respondents were female, were aged 50–59, had a university education, and had a good or average health status. Most of their job category were the first-line caregiver, had 1–5 years of experience in the field of LTCF, and served more than 16 elderly people per day. The average monthly salary was New Taiwan Dollar 30,000-$49,999. Regarding COVID-19 care experience, most of the respondents did not have experience caring for suspected or confirmed COVID-19 patients.

### Distribution of COVID-19 health literacy

**Fig. 1:**
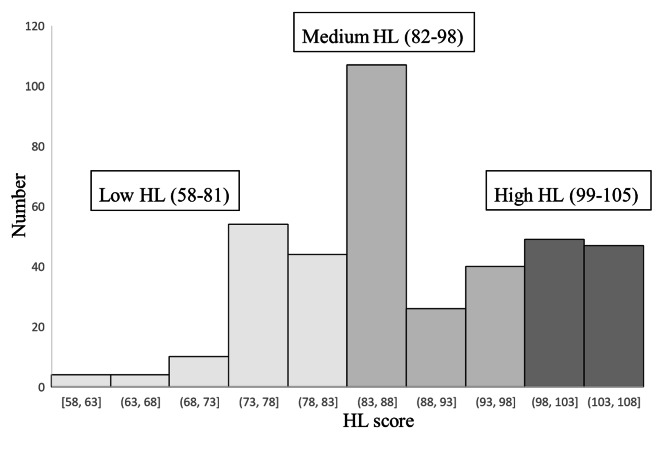
Distribution of staff COVID-19 health literacy scores (N = 385)

The results of the COVID-19 health literacy distribution are shown in Table 3. Questions 1 through 7 evaluated the ability of the staff of the elderly welfare organizations to obtain information about COVID-19, questions 8 through 14 assessed their ability to understand COVID-19 information, and questions 15 through 21 were designed to measure their ability to apply COVID-19 information based on their ability to implement the New Life campaign to eliminate the COVID-19 epidemic. Overall, COVID-19 health literacy had a mean score of 88.7 ± 10.4 and a range of 58–105. Finally, the total COVID-19 health literacy was calculated using quartiles as shown in Fig. 1. The respondents were further categorized into low-, medium-, and high-level COVID-19 health literacy groups according to their scores. According to the results, scores from 58 to 81 indicated a low level of health literacy (23.9%), 82–98 indicated a medium level of health literacy (49.3%), and 99–105 indicated a high level of COVID-19 health literacy (26.8%).

### COVID-19 Health literacy – single-variable analysis

The results of the single-variable analysis of demographic variables and COVID-19 health literacy are shown in Table 4. The results showed that the total COVID-19 health literacy score differed significantly by education level, while the results for the other variables did not reach statistical significance. In the section on job characteristics (Table 5), significant differences in the total COVID-19 health literacy score were found for job category (p = 0.001) and number of persons served per day (p = 0.01), while no significant differences were found for the other variables. The total COVID-19 health literacy scores were significantly greater for nursing staff than for nursing aides and significantly greater for those who served 16 or more persons per day than for those who served 1–10 persons.

Table 6 presents the analysis on COVID-19 services and prevention. The results showed that significant differences were found for infectious disease-related training, with those who had received training being more likely to have higher COVID-19 health literacy scores than their counterparts (p < 0.001); however, no significant differences were found for the remaining variables.

### Logistic regression analysis of COVID-19 health literacy level

The results of the logistic regression analysis of health literacy level are shown in Table 7. The demographic variables, work characteristics, COVID-19 services and prevention education were analyzed in a logistic regression model with health literacy level (> 82 vs. ≤82). The results showed that the job category (nurse practitioner vs. caregiver, OR = 7.25, 95% CI = 2.46–21.44), experience in caring for confirmed COVID-19 patients (yes vs. no, OR = 0.13, 95% CI = 0.02–0.98), and training related to infectious disease prevention and control (yes vs. no, OR = 2.8, 95% CI = 1.52–5.15) were significantly associated with a higher level of COVID-19 health literacy.

## Discussion

### COVID-19 health awareness of the sample

Public health literacy and communication of COVID-19 have proven to be a prerequisite for effective communication as part of the control strategy [17], and focusing on health literacy could thus be an essential strategic intervention yielding long-term benefits [18]. COVID-19 infection in institutionalized people is associated with the infection rate in nursing home workers [19]. Several public health interventions and health policy strategies, adequate resources, and focused clinical quality improvement initiatives can help calm the COVID-19 pandemic within LTCF [20]. In this study, the COVID-19 health literacy scale for workers in elderly welfare organizations consisted of 21 questions, with a total score ranging from 21 to 105. Overall, 76.1% of the staff had adequate health literacy; the question “I can get information about prevention of COVID-19 infection” received the most positive rating. In a previous study in a Chinese hospital of staff knowledge and attitudes toward COVID-19, 89.51% of staff had a broad understanding of COVID-19 [21]. Since January 2020, COVID-19-related information, such as information on real-time outbreaks and related outbreak events, has been broadcasted continuously through government agencies, mass media, print reports, and online social media. During the COVID-19 epidemic, health literacy has become an important competency. Previous study results showed that both adequate knowledge and positive attitudes were sufficient to slow the spread of COVID-19 [22]. Another study of health literacy differences in COVID-19 knowledge, attitudes, beliefs, and behaviors among Australian residents showed that people with inadequate health literacy had poorer understanding of COVID-19 symptoms and infection prevention behaviors and were less likely to find information about COVID-19 and understand government information than those with adequate health literacy [12]. However, the health literacy profile of domestic elderly welfare workers, who are at higher risk for infection, appears to be poorly understood; therefore, this study aimed to explore this area and provide the first empirical insights in Taiwan.

### Analysis of factors related to COVID-19 health literacy

The results of this study are consistent with a study of health care workers’ knowledge concerns and attitudes toward the COVID-19 pandemic in Saudi Arabia, which showed that individuals with a college education and above were more concerned about COVID-19 messages than those with basic diplomas. In addition, this study is consistent with a Chinese study of health literacy among outpatient nurses during the COVID-19 period, which showed that most outpatient nurses had high levels of health literacy [23]. Nursing staff were more concerned about COVID-19 messages than other medical staff or technicians, indicating that more educated nursing staff in their job category had a relatively higher level of access to COVID-19 messages, which helped to improve their COVID-19 health literacy.

In addition, the total score of COVID-19 health literacy was significantly greater for those who served more than 16 people per day than for those who served 1–10 people per day, indicating that those who served more people per day were more concerned about COVID-19; this is inferred to be the reason for caring for more patients, which helps to improve COVID-19 health literacy. Another previous study also showed that direct contact with patients with a confirmed COVID-19 diagnosis was positively associated with their access to COVID-19 information, and that their increased knowledge of COVID-19 information contributed to improved COVID-19 health literacy in the work service [24]. However, the present study found that the health literacy of workers who had cared for suspected or confirmed COVID-19 patients was lower than that of workers who had no caregiving experience; this may be because caregivers were mostly front-line workers or were assigned to this caregiving task by the organization, which was less associated with their health literacy. The front-line workers tend to be less educated or older than other staff in long-term care organizations. However, the age or educational level of participants were not significantly associated with COVID-19 health literacy level in this study. It is needed to focus these factors on the effects toward pandemic diseases in the future study. In addition, COVID-19 health literacy was lower among staff with longer monthly service hours, probably because they were mostly frontline staff and had lower health literacy than other professionals. Finally, the COVID-19 health literacy level of those who had received infectious disease prevention and control-related training in this study was significantly greater than that of those who had not received infectious disease prevention and control-related training; good infectious disease prevention and control-related training strengthens workers’ ability to obtain, understand, and apply COVID-19 messages.

COVID-19 awareness is significantly associated with health literacy [25], therefore, addressing the health literacy, language and cultural needs of the community in public health messaging about COVID-19 must now be a priority [12]. Health literacy and COVID-19 knowledge could be improved using diverse information sources, such as using digital media and face-to-face communication [26]. In long term care facilities, the employers have the means to mediate public health decision-making by implementing educational programs and offering incentives to raise awareness towards COVID-19 prevention [27]. Therefore, the health literacy of workers can be enhanced when their ability to care for COVID-19 is strengthened [28].

The main limitations of this study include the use of convenience sampling for data collection, which does not represent the true picture of COVID-19 health literacy among all workers in LTCF. There is not golden standard of the COVID-19 health literacy in the previous literature as we know. Therefore, we objectively used quartiles of COVID-19 health literacy scores as low- (25%), medium- (50%), and high-level (75%) groups. In addition, changes in the epidemic caused a limitation of the study instrument; that is, the questionnaire was not updated immediately. Furthermore, the collection process coincided with the peak of the epidemic, and respondents’ work pressure affected their willingness to complete the questionnaire. In addition, the survey was designed as a cross-sectional study, which could only provide an understanding of workers’ COVID-19 health literacy and reveal the factors related to it but could not establish the influencing factors.

## Conclusion

The main purpose of this study was to understand the disparities in COVID-19 health literacy and related factors among staff working in LTCF with different job titles and characteristics. In the single-variable analysis, there were significant differences based on the demographic variables (education, type of job category, number of persons served per day, and educational training in infectious disease prevention and control) and COVID-19 health literacy. Based on a multivariate logistic regression analysis, controlling for the relevant variables, there were significant associations between type of job category, number of service hours per month, experience in caring for suspected or confirmed COVID-19 patients, and training in infectious disease prevention and control, and the level of COVID-19 health literacy. These findings could be used to help policy makers improve the current COVID-19 health literacy policy for caregivers and develop strategies by institutional stakeholders to reduce barriers to obtaining COVID-19-related information. This study also recommends that organizations provide up-to-date information on COVID-19 to their staff, especially frontline caregivers, and strengthen COVID-19 infection control education and training for all staff to eliminate the variability in health literacy.

**Table 1:** Sample demographic characteristics (N = 385)

Variables	n	%	Mean ± S.D.(Range)
**Gender**			
Male	78	20.3	
Female	307	79.7	
**Age (years old)**			46.2 ± 12.2(34-58.4)
20–29	47	12.2	
30–39	68	17.7	
40–49	97	25.2	
50–59	122	31.7	
60–69	45	11.7	
70–79	6	1.6	
**Education**			
Primary School	15	3.9	
Middle School	47	12.2	
High School	83	21.6	
College	56	14.5	
University	150	39.0	
Graduate School	34	8.8	
**Marital status**			
Unmarried	134	34.8	
Married	211	54.8	
Widowed	19	4.9	
Divorced	21	5.5	
**Religious beliefs**			
None	149	38.7	
Buddhism	113	29.4	
Taoism	66	17.1	
Christianity	47	12.2	
Catholicism	6	1.6	
Kuan Tao	4	1.0	
**Are you the primary household income earner?**			
Yes	202	52.5	
No	183	47.5	
**Self-perceived health status**			
Very good	55	14.3	
Good	168	43.6	
Normal	148	38.4	
Poor	13	3.4	
Very poor	1	0.3	

**Table 2:** Sample work characteristics (N = 385)

Variables	n	%	Mean ± S.D (Range)
Job category (multiple choice)			
Nursing aide	184	47.8	
Nurse	99	25.7	
Administrative staff	38	9.9	
Social worker	32	8.3	
Supervisor	17	4.4	
Physiotherapist	5	1.3	
Manager	3	0.8	
Nutritionist	3	0.8	
Occupational therapist	3	0.8	
Pharmacist	1	0.3	
Professional certificate			
Nursing aide (general caregiver)	197	51.2	
Nurse	101	26.2	
Social worker	17	4.4	
Other*	49	12.7	
None	21	5.5	
Years since professional qualification obtained (year)			4.8 ± 1.8(3-6.6)
< 1	41	10.6	
≧1 - <2	29	7.5	
≧2 - <3	26	6.8	
≧3 - <4	23	6.0	
≧4 -< 5	34	8.8	
≧5	232	60.3	
Total years of service in the field of long-term care			7.9 ± 8.1(-0.2-16)
1–4	184	47.8	
5–9	74	19.2	
10–14	60	15.6	
15–19	29	7.5	
20–24	15	3.9	
25–29	10	2.6	
≧30	13	3.4	
Total years of service at the current institution			6.8 ± 8.1(0-14.9)
1–4	217	56.4	
5–9	71	18.4	
10–14	43	11.2	
15–19	17	4.4	
20–24	14	3.6	
25–29	11	2.9	
≧30	12	3.1	
Number of people served per day			
1–4 people	25	6.5	
5–10 people	80	20.8	
11–15 people	37	9.6	
≧16 people	139	36.1	
Indirect services	104	27.0	
Monthly service hours			
40–79 h	38	9.9	
80–119 h	17	4.4	
120–159 h	39	10.1	
≧160 h	291	75.6	
Monthly salary (NTD)			
Under $29,999	61	15.8	
$30,000-$49,999	243	63.1	
More than $50,000	81	21.0	

*(Executives, supervisors, managers, nutritionist, physical therapists, occupational therapists, pharmacists, drivers, cleaners, security, accountants)

**Table 3:** Distribution of COVID-19 health literacy responses among workers (N = 385)

Variable Name	Strongly agree	Agree	Neutral	Disagree	Strongly disagree
	n(%)	n(%)	n(%)	n(%)	n(%)
1. I can get information about prevention of COVID-19 infection.	159(41.3)	219(56.9)	6(1.6)	1(0.3)	0
2. I can get information about the organization’s COVID-19 prevention & management guidelines.	155(40.3)	217(56.4)	12(3.1)	1(0.3)	0
3. I can get information about the symptoms of contracting COVID-19.	153(39.7)	220(57.1)	11(2.9)	1(0.3)	0
4. I can get information about COVID-19 testing at my nearest medical facility.	113(29.4)	205(53.2)	49(12.7)	12(3.1)	6(1.6)
5. I have access to information about supportive care for patients with COVID-19.	97(25.2)	166(43.1)	106(27.5)	12(3.1)	4(1)
6. I can get information about self-health management, home isolation and home quarantine.	123(31.9)	199(51.7)	59(15.3)	2(0.5)	2(0.5)
7. I can get information about the New Life Movement as the COVID-19 pandemic slows down.	191(49.6)	157(40.8)	37(9.6)	0	0
8. I can understand the side effects and protective effects of various COVID-19 vaccines.	135(35.1)	221(57.4)	26(6.8)	3(0.8)	0
9. I can understand the current government precautions against COVID-19.	144(37.4)	215(55.8)	21(5.5)	4(1)	1(0.3)
10. I can understand the symptoms of COVID-19.	131(34)	216(56.1)	36(9.4)	2(0.5)	0
11. I can understand the appointment method for COVID-19 testing at the medical facility.	107(27.8)	214(55.6)	53(13.8)	10(2.6)	1(0.3)
12. I can understand supportive care for patients with COVID-19.	108(28.1)	177(46)	83(21.6)	14(3.6)	3(0.8)
13. I can understand the significance of autonomous health management, home isolation and home quarantine.	150(39)	189(49.1)	42(10.9)	3(0.8)	1(0.3)
14. I can understand the New Life Movement as the COVID-19 epidemic slows down.	185(48.1)	167(43.4)	33(8.6)	0	0
15. I can judge whether media reports on COVID-19 precautions can be trusted.	121(31.4)	196(50.9)	65(16.9)	3(0.8)	0
16. I can properly use all protective equipment to avoid contracting COVID-19.	124(32.2)	224(58.2)	34(8.8)	3(0.8)	0
17. I can judge whether measures to prevent COVID-19 infection are effective.	135(35.1)	217(56.4)	32(8.3)	1(0.3)	0
18. I can make an appointment for a COVID-19 test myself at a medical facility.	141(36.6)	209(54.3)	31(8.1)	4(1)	0
19. I can self-use COVID-19 rapid test kits.	122(31.7)	181(47)	52(13.5)	25(6.5)	5(1.3)
20. I can implement the New Life Movement as the COVID-19 epidemic slows down.	206(53.5)	164(42.6)	14(3.6)	1(0.3)	0
21. I can judge the difference between autonomous health management, home isolation and home quarantine.	133(34.5)	208(54)	39(10.1)	5(1.3)	0
* Average of COVID-19 health literacy total score	88.7 ± 10.4 (range: 58–105)				

*Total score of COVID-19 health literacy: a total of 21 questions, with a score range of 21–105; the higher the score is, the better the COVID-19 health literacy.

**Table 4:** Univariate analysis of demographic characteristics and COVID-19 health literacy (N = 385)

Variables	n	Mean ± S.D	t or F^*^	P	postmortem
Gender			1.175	0.241	
Male	78	89.94 ± 10.49			
Female	307	88.38 ± 10.39			
Age			0.936	0.423	
20–35	35	91.2 ± 10.53			
36–50	138	87.99 ± 10.90			
51–64	144	88.94 ± 10.37			
≧65	68	88.34 ± 9.41			
Education			4.064	0.003	3 > 1
1. Below middle school	47	85.24 ± 9.75			
2. High school	83	87.57 ± 10.01			
3. College or above	240	89.98 ± 10.51			
Marital status			-0.448	0.655	
Unmarried	174	89.44 ± 9.96			
Married	251	88.91 ± 10.79			
Religious beliefs			0.246	0.864	
None	149	88.15 ± 11.01			
Buddhism	113	88.96 ± 9.68			
Christianity	47	88.87 ± 10.38			
Other	76	89.29 ± 10.45			
Are you the primary household income earner?			-0.667	0.505	
Yes	202	88.36 ± 10.66			
No	183	89.07 ± 10.15			
Self-perceived health status			2.101	0.124	
Healthy	223	89.62 ± 10.38			
Normal	148	87.43 ± 10.39			
Unhealthy	14	87.43 ± 10.34			

^*^The scores for gender, main income earner and perceived health status were compared by t test, and the other groups were compared by one-way ANOVA.

**Table 5:** Univariate analysis of job characteristics and COVID-19 health literacy (N = 385)

Variables	n	Mean ± S.D	t or F^*^	P	Post test
Job category			5.237	0.001	
1. Nursing aide	184	86.91 ± 10.69			
2. Nurse	99	92.00 ± 9.29			2 > 1
3. Social worker	32	88.35 ± 9.09			
4. Other	70	88.99 ± 10.73			
Professional certificate			-0.443	0.658	
Yes	336	88.79 ± 10.41			
No	49	88.08 ± 10.50			
Years since professional qualification obtained			0.050	0.951	
< 1	41	89.07 ± 8.77			
1–5	112	88.49 ± 10.98			
> 5	232	88.73 ± 10.44			
Total years of service in the field of long-term care			0.290	0.749	
<10	258	88.68 ± 10.48			
10–20	89	88.28 ± 10.31			
> 20	25	89.82 ± 10.42			
Total years of service at the current institution			0.229	0.795	
<10	288	88.88 ± 10.54			
10–20	60	87.88 ± 10.04			
> 20	25	88.59 ± 10.41			
Number of people served daily			3.835	0.010	
1. 1–10 people	105	85.94 ± 10.08			3 > 1
2. 11–15 people	37	90.22 ± 10.54			
3. ≧16 people	139	90.25 ± 10.39			
4. Indirect service	104	88.87 ± 10.32			
Monthly service hours			0.660	0.577	
40–79 h	38	89.82 ± 8.52			
80–119 h	17	89.59 ± 11.62			
120–159 h	39	86.72 ± 9.78			
≧160 h	291	88.77 ± 10.66			
Monthly salary (NTD)			2.315	0.100	
Below $ 29,999	61	88.95 ± 9.82			
$30,000- $49,999	243	87.94 ± 10.42			
$50,000- $69,999	81	90.80 ± 10.65			

^*^The t test was used to compare the scores of professional certificates and work service areas, while the other groups were compared by one-way ANOVA.

**Table 6:** Univariate analysis of infectious disease prevention experience and COVID-19 health literacy (N = 385)

Variables	n	Mean ± S.D	t test	P
Experience in caring for suspected and confirmed COVID-19 patients			0.441	0.659
Yes	20	89.70 ± 11.136		
No	365	88.64 ± 10.39		
Experience in caring for confirmed COVID-19 patients			-0.116	0.907
Yes	17	88.41 ± 12.44		
No	368	88.71 ± 10.33		
Vaccinated against COVID-19			-2.017	0.165
Yes	382	88.66 ± 10.45		
No	3	93.00 ± 3.61		
Received training in the prevention and treatment of infectious diseases			3.745	< 0.001
Yes	243	90.19 ± 10.37		
No	142	86.14 ± 10.02		

**Table 7:** Logistic regression analysis of COVID-19 health literacy level (> 82 points vs. ≤82 points) (N = 385)

Variables^*^	O.R.	95% C.I.	P
Gender (ref: Female)	1.00		
Male	2.46	1.15–5.26	0.020
Job category (ref: Nursing aide)	1.00		
Nurse	7.25	2.46–21.44	< 0.001
Social worker	1.04	0.32–3.44	0.942
Other	1.08	0.41–2.85	0.880
Service hours per month (ref: 40–79)	1.00		
80–119 120–159 ≧ 160	0.36 0.30 0.26	0.06–2.17 0.06–1.37 0.07–0.97	0.265 0.120 0.044
Cared for a confirmed COVID-19 patient (ref: No)	1.00		
Yes	0.13	0.02–0.98	0.047
Received training related to the prevention and treatment of infectious diseases (ref: No)	1.00		
Yes	2.80	1.52–5.15	0.001

^*^Variables with no significant difference: age, education, marital status, religious beliefs, primary household income earner, self-perceived health status, length of professional certification, total years of service in long-term care, total years of service in current institution, number of persons served per day, monthly salary, care of suspected or confirmed COVID-19 patients, and COVID-19 vaccination.

O.R.: odds ratio

C.I.: confidence interval

## Data Availability

The authors confirm that the data supporting the findings of this study are available on request from the corresponding author.

## References

[1] Pirrie M, Agarwal G (2021). Older adults living in social housing in Canada: the next COVID-19 hotspot?. Can J Public Health.

[2] Taiwan Ministry of Health and Welfare. The conditions of elderly long-term care and caring institutions. https://www.mohw.gov.tw/dl-22134-14969240-e876-4061-b76d-bf1bf2efd4ed.html. Accessed 6 August 2022.

[3] Miralles O, Sanchez-Rodriguez D, Marco E, Annweiler C, Baztan A, Betancor É (2021). Unmet needs, health policies, and actions during the COVID-19 pandemic: a report from six european countries. Eur Geriatr Med.

[4] Wang J, Yang W, Pan L, Ji JS, Shen J, Zhao K. er al. Prevention and control of COVID-19 in nursing homes, orphanages, and prisons. Environ Pollut. 2020;266:115161. 10.1016/j.envpol.2020.115161. 10.1016/j.envpol.2020.115161 PMC7332257 32645554

[5] Taiwan Ministry of Health and Welfare. Guidelines for infection control in healthcare facilities. https://www.cdc.gov.tw/Category/MPage/I92jtldmxZO_oolFPzP9HQ. Accessed 6 August 6 2022.

[6] Ishikawa H, Kato M, Kiuchi T (2021). Declines in health literacy and health-related quality of life during the COVID-19 pandemic: a longitudinal study of the japanese general population. BMC Public Health.

[7] Lin CC, Sung HY, Chuang CM, Lan YH (2009). Knowledge, attitude and behavior of infection control among staff in long-term care facilities in central Taiwan. J Long Term Care.

[8] Mani NS, Ottosen T, Fratta M, Yu F (2021). A health literacy analysis of the consumer-oriented COVID-19 information produced by ten state health departments. J Med Libr Assoc.

[9] Baker DW (2006). The meaning and the measure of health literacy. J Gen Intern Med.

[10] Chao KI, Wang SC, Huang TC, Woung LC, Lee OK, Chu D, et al. The current status of health literacy promotion in Taipei City Hospital. Taipei City Medical Journal. 2017;14. 10.6200/TCMJ.2017.14.2.12. 224 – 33.

[11] Naveed MA, Shaukat R (2022). Health literacy predicts Covid-19 awareness and protective behaviours of university students. Health Info Libr J.

[12] McCaffery KJ, Dodd RH, Cvejic E, Ayrek J, Batcup C, Isautier JM (2020). Health literacy and disparities in COVID-19-related knowledge, attitudes, beliefs and behaviours in Australia. Public Health Res Pract.

[13] Ishizuka-Inoue M, Shimoura K, Nagai-Tanima M, Aoyama T (2023). The relationship between health literacy, knowledge, fear, and COVID-19 prevention behavior in different age groups: cross-sectional web-based study. JMIR Form Res.

[14] Choi HS, Lee JE (2023). Factors affecting COVID-19 preventive behaviors of young adults based on eHealth literacy and the Health Belief Model: a cross-sectional study. Inquiry.

[15] Taiwan Ministry of Health and Welfare. Number of workers in elderly long term care, nursing and caring institutions. https://dep.mohw.gov.tw/dos/cp-5223-62358-113.html#_4.%E9%95%B7%E6%9 C%9F%E7%85%A7%E9%A1%A7. Accessed 20 August 2021.

[16] Raosoft Inc. Sample size calculator. Retrieved from http://www.raosoft.com/samplesize.html. Accessed 20 November 2021.

[17] Hange N, Agoli AM, Pormento MKL, Sharma A, Somagutta MR, Paikkattil N et al. Impact of COVID-19 response on public health literacy and communication. Health Promot Perspect. 2022;12(1):1–9. 10.34172/hpp.2022.01. eCollection 2022. 10.34172/hpp.2022.01 PMC9277294 35854843

[18] Gautam VSD, Rustagi N, Mittal A, Patel M, Shafi S, Thirunavukkarasu P (2021). Health literacy, preventive COVID 19 behaviour and adherence to chronic disease treatment during lockdown among patients registered at primary health facility in urban Jodhpur, Rajasthan. Diabetes Metab Syndr.

[19] Burgaña Agoües A, Serra Gallego M, Hernández Resa R, Joven Llorente B, Lloret Arabi M, Ortiz Rodriguez J (2021). Risk factors for COVID-19 morbidity and mortality in institutionalised elderly people. Int J Environ Res Public Health.

[20] Ouslander JG, Grabowski DC (2020). COVID-19 in nursing homes: calming the perfect storm. J Am Geriatr Soc.

[21] Shi Y, Wang J, Yang Y (2020). Knowledge and attitudes of medical staff in chinese psychiatric hospitals regarding COVID-19. Brain Behav Immun Health.

[22] Alemu T, Amare S, Legesse S, Abera A, Ayalew M, Bezabih B (2021). COVID-19 knowledge, attitude, practices and their associated factors among Dessie city residents, northeast Ethiopia: a cross-sectional study. Risk Manag Healthc Policy.

[23] Rohwer E, Mojtahedzadeh N, Neumann FA, Nienhaus A, Augustin M, Harth V (2021). The role of health literacy among outpatient caregivers during the COVID-19 pandemic. Int J Environ Res Public Health.

[24] Abolfotouh MA, Almutairi AF, BaniMustafa AA, Hussein MA (2020). Perception and attitude of healthcare workers in Saudi Arabia with regard to COVID-19 pandemic and potential associated predictors. BMC Infect Dis.

[25] Chen C, Xu T, Chen Y, Xu Y, Ge L, Yao D (2022). Does health literacy promote COVID-19 awareness? Evidence from Zhejiang, China. Front Public Health.

[26] Inoue M, Shimoura K, Nagai-Tanima M, Aoyama T (2022). The relationship between information sources, health literacy, and COVID-19 knowledge in the COVID-19 infodemic: cross-sectional online study in Japan. J Med Internet Res.

[27] Ruf AK, Völkl-Kernstock S, Eitenberger M, Gabriel M, Klager E, Kletecka-Pulker M (2022). Employer impact on COVID-19 vaccine uptake among nursing and social care employees in Austria. Front Public Health.

[28] Kanjee Z, Catterick K, Moll AP, Amico KR, Friedland GH (2011). Tuberculosis infection control in rural South Africa: survey of knowledge, attitude and practice in hospital staff. J Hospl Infect.

